# GastroHUN an Endoscopy Dataset of Complete Systematic Screening Protocol for the Stomach

**DOI:** 10.1038/s41597-025-04401-5

**Published:** 2025-01-17

**Authors:** Diego Bravo, Juan Frias, Felipe Vera, Juan Trejos, Carlos Martínez, Martín Gómez, Fabio González, Eduardo Romero

**Affiliations:** 1 Universidad Nacional de Colombia, Bogotá, 1100111 Colombia; 2 Computer Imaging and Medical Applications Laboratory (CIM@LAB), Bogotá, 1100111 Colombia; 3 Universidad Nacional de Colombia, Medicina Interna, Bogotá, 1100111 Colombia; 4 Hospital Universitario Nacional de Colombia, Gastroeneterology, Bogotá, 1100111 Colombia; 5 Machine Learning, Perception and Discovery Lab (MindLab), Bogotá, 1100111 Colombia

**Keywords:** Oesophagogastroscopy, Stomach, Stomach diseases

## Abstract

Endoscopy is vital for detecting and diagnosing gastrointestinal diseases. Systematic examination protocols are key to enhancing detection, particularly for the early identification of premalignant conditions. Publicly available endoscopy image databases are crucial for machine learning research, yet challenges persist, particularly in identifying upper gastrointestinal anatomical landmarks to ensure effective and precise endoscopic procedures. However, many existing datasets have inconsistent labeling and limited accessibility, leading to biased models and reduced generalizability. This paper introduces GastroHUN, an open dataset documenting stomach screening procedures based on a systematic protocol. GastroHUN includes 8,834 images from 387 patients and 4,729 labeled video sequences, all annotated by four experts. The dataset covers 22 anatomical landmarks in the stomach and includes an additional category for unqualified images, making it a valuable resource for AI model development. By providing a robust public dataset and baseline deep learning models for image and sequence classification, GastroHUN serves as a benchmark for future research and aids in the development of more effective algorithms.

## Background & Summary

Stomach gastric cancer is one of those oncologic processes with the poorest prognosis and yet it can go undetected during routine examinations. Unfortunately, current methods often fail to identify premalignant lesions and early-stage cancers, thereby limiting treatment options and patient survival rates. According to the International Agency for Research on Cancer (IARC) https://gco.iarc.fr/today/en/fact-sheets-cancers, the specialized cancer agency of the World Health Organization (WHO), stomach cancer remains a significant global public health concern. In 2022, IARC estimated 968,784 new cases and 660,175 deaths attributable to stomach cancer worldwide^1^. Esophagogastroduodenoscopy (EGD) is the screening procedure for diagnosing upper gastrointestinal (GI) diseases and upper GI cancers in high-risk areas^2^.

**Fig. 1:**
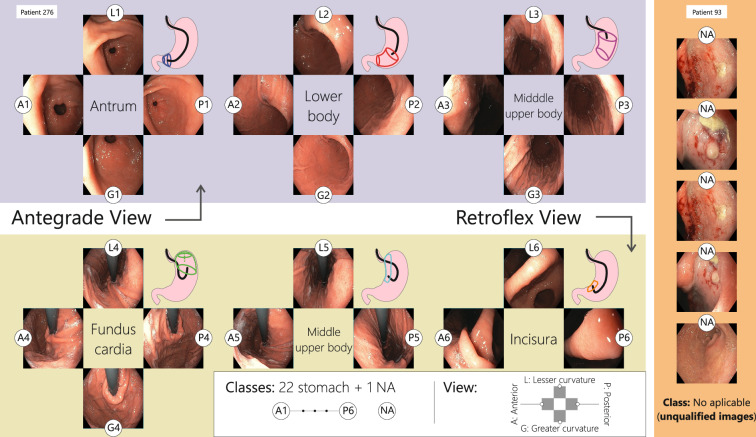
Photographic documentation protocol of the stomach that begins as soon as the endoscope is inserted into the gastric antrum. With the anterograde view, endoscopic photographs of 4 quadrants of the gastric antrum, body, and upper middle body are taken. Then, with the retroflex view, endoscopic photographs of 4 quadrants of the fundus cardia, and 3 quadrants of the upper middle body and gastric incisura are taken. The SSS series consists of 22 endoscopic photographs of the stomach. Images where the intended category is not clearly visible, or a documented lesion is present are categorized as “NA”. The abbreviations are L for lesser curvature, A for anterior wall, G for greater curvature, and P for posterior wall.

A main purpose of the EGD screening setting is to enhance the detection rate of early-stage gastric cancers (EGC) and to reduce cancer-related mortality, both tasks highly dependent on the operator’s expertise. In fact, 20%–25% of EGC are missed^3^ while 11.3% of upper gastrointestinal cancers in more advanced stages are not detected^4^. Certain locations, the cardias, body lesser curvature or posterior wall, have been reported as gastric regions with higher risk of lesions to be missed^5,6^. Therefore, accurate diagnosis relies on exhaustive scanning of the gastric mucosa^7^, which should be documented with photographs during endoscopic procedure, ensuring exploration is complete. Several protocols worldwide have been introduced to visually register the explored areas of the upper gastrointestinal tract, differing among them in the specific areas to be documented. Currently, the European Society of Gastrointestinal Endoscopy (ESGE) proposed that photodocumentation in a normal endoscopic examination should have at least 10 gastric regions^8^, The Korean Society of Gastrointestinal Endoscopy (KSGE) recommends photodocumentation of at least 8 gastric regions in a normal EGD examination, with additional photos of suspicious lesions^9^. In Japan, the “Systematic Screening Protocol for the Stomach (SSS)”, suggest 22 gastric regions^10^ (see protocol in Fig. 1). Although all these protocols have shown to be useful, the Japanese strategy has shown to be more effective in reducing mortality rates.

The gastroenterology community agrees about the importance of auditing these procedures, thinking effectiveness of these procedures could be benefited by ensuring the protocol is strictly followed^11^. However, in actual clinical scenarios this audit sounds unrealistic, except if a part or the entirety of the audit procedure is automated. Artificial Intelligence (AI) systems present a real opportunity for implementing automatic audits. However, realizing this potential requires two key improvements in data: a significant increase in the amount of relevant data and enhanced accessibility to existing datasets. AI systems are fundamentally dependent on data, and their performance generally improves with larger datasets. A growing number of data collections demonstrate the feasibility of automated audits. However, most of these data repositories remain private. It is important to note that data protection decisions are often guided by medical ethics committees, as acquiring health data poses unique challenges not commonly found in non-medical machine learning fields.

Medical imaging databases are crucial for advancing algorithms in medical image analysis, particularly in deep learning applications across a wide range of clinical domains. Initiatives like the Alzheimer’s Disease Neuroimaging Initiative (ADNI)^12,13^, the Human Brain Connectivity Database (HBCD)^14,15^, and the Cancer Genome Atlas (TCGA)^16^ are just a few examples of the invaluable data repositories that have fueled progress in neuroscience^17^, digital pathology, and other fields^18^. Beyond these examples, there are also expansive databases supporting research in areas such as radiology^19^, cardiology^20^, retinal^21^, and musculoskeletal imaging, all of which have become essential tools for developing transformative AI technologies to enhance clinical decision-making and patient care. However, there is a notable disparity in accessible, large-scale gastrointestinal (GI) datasets. Most GI datasets are private, limited in size, and primarily focus on lower GI tract abnormalities. Notable public endoscopy datasets include Endomapper, which provides annotated videocolonoscopy procedures^22^, Kvasir-Capsule, featuring small bowel images in 14 categories^23^, and HyperKvasir, which offers comprehensive data on the upper and lower GI tracts^24^. Despite these efforts, the scarcity of large-scale, accessible GI datasets, particularly for the upper GI tract and normal cases, remains a significant challenge. There is currently no public collection of upper GI videoendoscopies that follow a standardized quality protocol for stomach screening. This highlights the urgent need for more extensive data-sharing initiatives in gastroenterology to support comprehensive research and algorithm development. Table 1 provides an overview of datasets containing photographic documentation of the stomach. All works, except for our own, were selected from a review by Renna, Francesco, *et al*.^25^. To determine if these databases were public or not, and under which conditions they might be accessed, authors were contacted via email and if after two-week no response was obtained, the corresponding collection was classified as private. Portions of the GastroHUN dataset have been used in previous works. Initially, 2,054 images were categorized into six classes by one medical expert (2023)^26,27^. The categories were later extended to 13 (2024)^28,29^. The dataset now includes a larger number of cases, 23 categories, diverse data types such as images, sequences, and videoendoscopies, contributions from multiple labelers, and fully adheres to a systematic detection protocol^10^.

**Table 1:** An overview of existing upper anatomical datasets, sorted by year of publication.

Dataset	Protocol	Classes	Size	Availability
Takiyama *et al*. (2018)^37^	Japanese Classification of Gastric Carcinoma	4 sites + 3 gastric sites	44,416 images	Private
Wu *et al*. (2019)^38^	SSS	10 or 26 (22 SSS + others)	24,549 images	Private
Xu *et al*. (2019)^39^	N/A	10 sites	75,275^*^ images	Private
Wu *et al*. (2019)^40^	SSS	26 sites (22 SSS + others) + NA	34,513 images; 107 sequences	Private
He *et al*. (2020)^41^	Modified British and Japanese guideline	11	3,704 images	By request
Igarashi *et al*. (2020)^42^	Unclear	10	85,246 images	Private
Chang *et al*. (2021)^43^	Unclear	8	15,723 images	Private
LI *et al*. (2021)^44^	SSS	7 non stomach + 24 gastric regions (22 SSS + others)	170,297 images; 5,779 sequences	Private
Choi *et al*. (2022)^45^	ESGE	8 sites	2,599 images	Private
Ding *et al*. (2021)^46^	Undefined	6 sites + 1 background	7,351 images	Private
Sun *et al*. (2022)^47^	Unclear	11	10,474 images	Private
Ours (GastroHUN, 2025)	SSS	22 SSS + NA	233 videoendoscopies; 8,834 images collected from 387 cases; 4,729 sequences derived from 223 cases	Public

^*^ including non-informative and NBI frames.

Contributions of this paper are as follows

- An open dataset is available at figshare^30^, containing 387 high-definition esophagogastroduodenoscopy cases recorded using the SSS Kenshi Yao protocol^10^. The dataset includes two types of recordings: 4,729 sequences from 223 videoendoscopies and 8,834 images, each representing a selected frame from one of the 22 stations of the Kenshi Yao protocol or unqualified category.
- Annotations were provided at the frame level by a panel of four experts: two recent graduates, and two final-year gastroenterology fellows. The specialist selected all representative samples during the procedure through image photodocumentation, the central frame of each sequence was chosen as the most representative. All frames and static images were labeled into one of 23 categories.
- A comprehensive validation framework for image and sequence classification is provided, encompassing data partitioning, performance metrics, and baseline models.

## Methods

### Use of human participants

The study adhered to the principles of the Declaration of Helsinki, and ethical approval was granted by the Ethics Committee of the Hospital Universitario Nacional de Colombia (approval number: CEI-2019-06-10). All patients signed an Informed Consent to Privacy Data Protection Authority, which explicitly allowed the use of their clinical and procedural data for research and educational purposes, including the development of computational methods to enhance diagnostic procedures for gastrointestinal diseases. Recordings were collected retrospectively from procedures scheduled between 2019 and 2023. Participants were informed that their information might be used to improve medical practice, with all data anonymized through the removal of metadata and renaming of files via a hash generator to ensure their identity cannot be traced. The Ethics Committee approved the publication of the dataset under an open license, considering the retrospective nature of the study, the informed consent provided, and the anonymization of the data, ensuring compliance with open-access requirements.

### Endoscopy procedure

The endoscopy procedure is carried out as follows: after scheduling for an upper gastrointestinal endoscopy, patients sign informed consent forms before entering the gastroenterology unit. Approximately 30 minutes before the procedure, they receive a preparation of 10 *mL* of a solution containing 400 *mg* of N-acetylcysteine and 200 *mg* of simethicone. Afterwards, patients lie on their left side during 5 minutes and then wait 20 to 30 minutes before the procedure. Once in the procedure room, a cannula is inserted into the patient’s right arm, and a certified anesthesiologist administers intravenous sedation with propofol. The patient standard posture during esophagogastroduodenoscopy (EGD) is the left lateral decubitus position. After sedation, an Olympus series 190 endoscope is introduced to aspirate gastric content residues, distend the cavity by injecting air and position it at the duodenum^31^.

After inspection of the duodenum with the monocular endoscope, a photographic record is performed as illustrated in Fig. 1. This photodocumentation starts at the pylorus’s position, after which gastroenterologists should perform the next exploration after the SSS Kenshi Yao protocol^10^:

1. The equipment is retracted 5 *cm* to initiate the antrum photo-documentation, beginning at the greater curvature and proceeding clockwise, capturing 4 overlapping photos: greater curvature (photo 1-G1), anterior wall (photo 2-A1), lesser curvature (photo 3-L1), and posterior wall (photo 4-P1).
2. The equipment is withdrawn 15 *cm* up to the distal gastric body, continuing clockwise to capture: greater curvature (photo 5-G2), anterior wall (photo 6-A2), lesser curvature (photo 7-L2), and posterior wall (photo 8-P2).
3. The equipment is then pulled back another 15 *cm* to the upper-middle gastric body, maintaining the clockwise documentation: greater curvature (photo 9-G3), anterior wall (photo 10-A3), lesser curvature (photo 11-L3), and posterior wall (photo 12-P3).
4. The gastroscope is advanced to the corporoantral junction where retroflexion is performed to visualize the cardias and gastric fundus regions. Photodocumentation proceeds: greater curvature (photo 13-G4), anterior wall (photo 14-A4), lesser curvature (photo 15-L4), and posterior wall (photo 16-P4).
5. Once the equipment is adjusted for rear view, the lesser curvature by 5 *cm* is fully exposed, capturing three additional photos: anterior wall (photo 17-A5), lesser curvature (photo 18-L5), and posterior wall (photo 19-P5).
6. Finally, after aligning the equipment tip for a complete view, the concluding photographs are the anterior wall (photo 20-A6), lesser curvature (photo 21-L6), and posterior wall (photo 22-P6).

### Recording endoscopy procedure and data

Data are herein presented either as single images or videos and were collected by standard endoscopy equipment: Olympus EVIS EXERA III CV-190 video processor, EVIS EXERA III CLV-190 light source and EXERA II TJF-Q180V and GFI-H170 gastroscope from the Department of Gastroenterology, Hospital Universitario Nacional de Colombia (HUN), in Bogotá (Colombia). HUN provides gastroenterology services to more than 4,000 patients per year. The procedures herein recorded were performed by 2 last year residents and two 2 gastroenterologists, and one master gastroenterologist with more than 20 years of experience and about 50,000 procedures following the SSS Kenshi Yao protocol^10^. The two residents of gastroenterology (FG - Team A) have documented an average of 500 procedures while gastroenterologists (G - Team B) have performed at least 1,000 procedures. Each case was independently annotated by experts from both Team A and Team B using a quadruple-blind labeling process. The images and videos were manually edited to remove any identifying information, such as direct and indirect identifiers and frames recorded when the camera was outside the patient’s body. Recordings were collected retrospectively from procedures scheduled between 2019 and 2023. At least one of the five gastroenterologists was present during recording sessions to ensure the quality of the acquisition without interfering with the medical procedures. The videos were recorded at 30 and 15 frames per second using video capture devices from either Epiphan (Ottawa, Canada, specializing in professional video capture hardware and audiovisual solutions) or Elgato (Corsair Components, Inc., Fremont, California, USA, specializing in consumer electronics and streaming peripherals) to capture footage from the endoscope.

## Data Records

The GastroHUN dataset is available at figshare^30^. Table 2 provides a summary of all data recorded within the dataset, which includes 8,834 annotated images and 4,729 annotated sequences. The dataset has a total size of 96.86 GB and is organized into three catalogs: “Labeled Images”, “Labeled Sequences”, and “Videoendoscopies”. The “Labeled Images” and “Labeled Sequences” catalogs contain archive files for each labeled class, while the “Videoendoscopies” catalog includes endoscopic findings and pathological diagnoses video files. An overview of the dataset structure is presented in Table 3.

**Table 2:** Overview of the data records in the GastroHUN dataset, which includes 387 patients.

Data Record	# Files	Description	Size (GB)
Labeled Images	8,834	22 anatomical landmark classes + NA	2.71
Labeled Sequences	4,729	22 anatomical landmark classes + NA	30.25
Videoendoscopies	237	from 233 procedures with diagnoses	63.90

The demographic distribution is as follows: Females have an average age of 63.2 ± 15.1 years (60%), and Males have an average age of 61.3 ± 16.4 years (40%).

**Table 3:** Detailed description of the columns of image and sequence metadata.

Dataset Information	
Column Name	Description
num_patient	Patient number (e.g., *7*)
filename	Unique image file named: 0c14fc9a-3781-4fa9-b8f3-1ece0af92ebd.jpg; Unique sequence file named: 0c14fc9a-3781-4fa9-b8f3-1ece0af92ebd.mp4
**Team Annotations**	
FG1 (Team A)	Annotation from Fellow Gastroenterology 1 - Team A (e.g., *A1*)
FG2 (Team A)	Annotation from Fellow Gastroenterology 2 - Team A (e.g., *A1*)
G1 (Team B)	Annotation from Gastroenterology 1 - Team B (e.g., *A1*)
G2 (Team B)	Annotation from Gastroenterology 2 - Team B (e.g., *A1*)
**Agreement Types**	
Complete	Indicates complete agreement across all annotations (e.g., *A1*)
Triple	Indicates agreement among three of the four annotations (e.g., *A1*)
FG	Indicates agreement between FG1 and FG2 annotations (e.g., *A1*)
G	Indicates agreement between G1 and G2 annotations (e.g., *A1*)
FG1-G1	Indicates agreement between FG1 (Team A) and G1 (Team B) (e.g., *A1*)
FG1-G2	Indicates agreement between FG1 (Team A) and G2 (Team B) (e.g., *A1*)
FG2-G1	Indicates agreement between FG2 (Team A) and G1 (Team B) (e.g., *A1*)
FG2-G2	Indicates agreement between FG2 (Team A) and G2 (Team B) (e.g., *A1*)

### Labeled Images

**Fig. 2:**
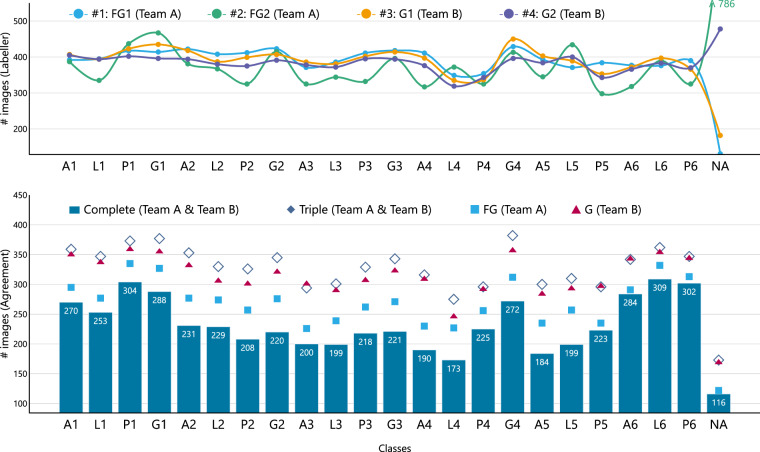
The distribution of images across different anatomical categories is shown by annotator and at various levels of agreement. “FG” stands for Fellow Gastroenterologist, and “G” stands for Gastroenterologist.

The dataset comprises 8,834 labeled images from 387 patients, with 8,053 images stored in JPG format, recorded by an Olympus MAJ-1925 portable memory provided with the Olympus EVIS EXCERA III CV-190 endoscope and whose default compression varies between 1/5 to 1/10 ratios. Additionally, 781 images stored in JPG format, captured as screenshots from recorded videoendoscopies. Figure 2 illustrates the 23 different classes representing the labeled images and the number of images in each class. A JSON file, **gastrohun-image-metadata.json** maps image filenames to their labels provided by the four gastroenterologists, including consensus labels across 8 levels, and features a column called *source_type* to specify whether each image is a direct endoscope capture or a video frame. The dataset’s key components are summarized in Table 3. The dataset offers a detailed overview of patient data, annotations from two different teams, and agreement labels to evaluate the consistency and reliability of the annotations. The category classes are organized following their location within the stomach, after the photodocumentation guidelines by SSS Kenshi Yao^10^. The image resolutions are distributed as follows: 8,427 images (91.16%) at [1080, 1350], and 407 images (8.84%) at [720, 900]. Frames extracted from video are available in two resolutions: 407 images at [720, 900] and 374 images at [1080, 1350]. Despite these variations, the set of experts did not perceive any difference among the different compression and resolution formats.

### Labeled Sequences

A sequence represents a ten-second video segment centered around an annotated frame, capturing five seconds before and after that frame for a particular station. When videoendoscopies are available, photodocumented images are used to extract temporal information within these sequences. The central frame of each sequence is assigned a label based on the Labeled Images process. The samples were generated using ffmpeg with the following settings: 15 frames per second (fps), encoded with libx264, pixel format yuv420p, baseline profile, and a bitrate of 23. All files are provided in MP4 format. The dataset includes 4,729 video sequences collected from 223 patients, obtained through standard recording procedures. A JSON file, **gastrohun-sequence-metadata.json**, maps each sequence to its corresponding label, independently assigned by four gastroenterologists with varying levels of agreement (see Table 3). The file also includes details such as the patient number, frame number, frames of the sequence, and the videoendoscopy name from the “Videoendoscopies” dataset. Sequence resolutions are distributed as follows: 4,043 sequences at [1080, 1350] and 686 sequences at [720, 900].

### Videoendoscopies

All files are in MP4 format, containing 237 videos from 233 patients. The video resolutions are distributed as follows: 206 endoscopies at [1080, 1350] and 31 at [720, 900]. Among these, 204 videos have a frame rate of 30 frames per second, while 33 have a frame rate of 15 frames per second. A JSON file, **gastrohun-videoendoscopy-metadata.json**, includes key diagnostic information in four columns. The *Diagnoses*, which lists conditions such as Chronic Gastritis, Peptic Esophagitis, and other related disorders. *Findings*, which describes observations from videoendoscopy procedures, *H. PYLORI*, indicating the infection status of Helicobacter pylori, and *OLGA*, which stages atrophic gastritis based on its severity according to the Operative Link for Gastritis Assessment (OLGA) system. This dataset offers a unique challenge for researchers due to the integration of videoendoscopies, allowing for a detailed analysis of not just images, but entire endoscopic sequence. This opens possibilities for tasks like classifying visual endoscopic findings or detecting the presence of Helicobacter pylori based on the visual examination of the gastric mucosa, as confirmed by pathology reports. Additionally, the dataset supports staging gastric conditions using OLGA, which can be valuable for early detection of premalignant conditions. A particularly compelling challenge is developing models that could: (1) predict metaplasia from white-light endoscopy videos, (2) quantify abnormal motility patterns in conditions that may be associated with Helicobacter pylori infection or OLGA stages, and (3) provide automatic quality assessment of complete upper gastrointestinal tract examinations. These tasks represent significant opportunities for advancing automated diagnostic tools in upper gastrointestinal disorders.

## Technical Validation

The technical quality of the GastroHUN dataset is ensured by evaluating inter-annotator agreement on image labels using Cohen’s kappa coefficient. This approach provides stratified data partitions and a validation framework for future research, enabling comparisons with existing image and sequence classification methods.

### Label Kappa Agreements

**Fig. 3:**
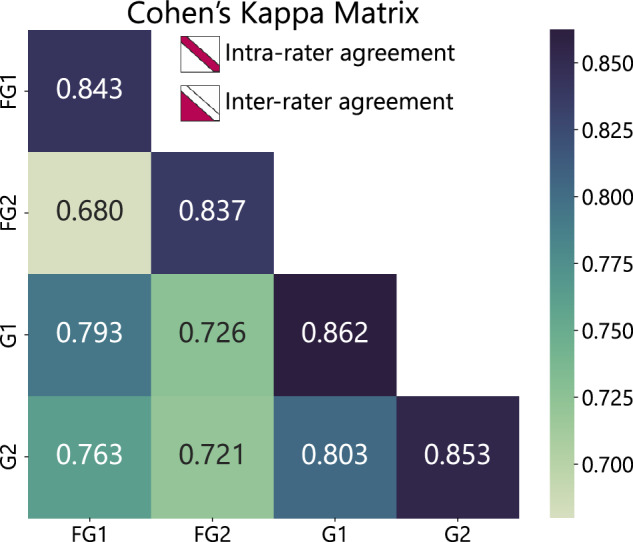
Cohen’s Kappa coefficients assess inter-rater (lower triangle) agreement among gastroenterologists and intra-rater (diagonal) consistency over time. “FG” denotes Fellow Gastroenterologists (Team A), and “G” refers to Gastroenterologists (Team B). The matrix illustrates temporal comparisons and paired annotators’ agreement.

In this study, labeling consistency among four raters was evaluated using the Cohen’s kappa coefficient which was pairwise computed to assess collective agreement: firstly 905 (9.761%) images were shown twice at different times and results are shown at the diagonal in Fig. 3, and secondly the lower triangle in Fig. 3 displays the agreement between pairs of experts across 8,834 images (100%). A thorough analysis of inter-rater agreement provides a deeper understanding of data integrity.

### Stratified data partition

**Fig. 4:**
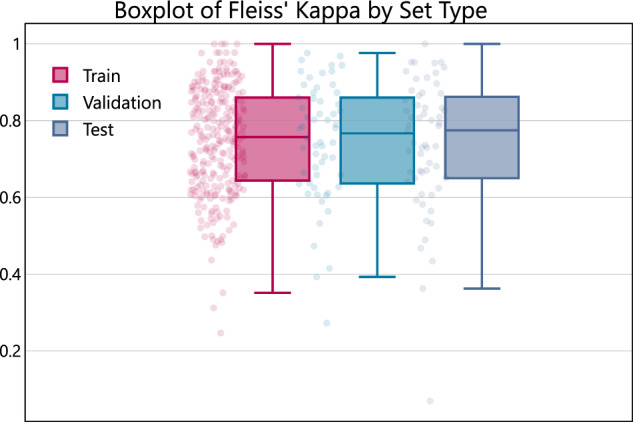
Boxplot illustrating the distribution of Fleiss’ Kappa coefficients across different patient sets. Each boxplot represents the inter-rater reliability within a specific set, highlighting the median, interquartile range, and potential outliers of the Kappa scores.

The dataset was divided using a stratified partition by patient, allocating 70% for training, 15% for validation, and 15% for testing. This technique ensures each subset shows similar label distribution with respect to the entire dataset. The stratification was performed using Fleiss’ Kappa to assess inter-rater agreement for each patient. Cases were subsequently divided into quartiles according to their Fleiss’ Kappa scores, and proportionally distributed across training, validation, and testing sets to ensure consistent distribution of agreement levels (refer to Fig. 4). The official splits are provided as CSV files in the data and code repository: **image_classification.csv** and **sequence_classification.csv**. These files follow the structure outlined in Table 3 and include a new column called *set_type*, which specifies the dataset type (e.g., *Train, Validation, or Testing*). These files ensure consistent use of the same cases for both image and sequence classification tasks.

### Machine Learning baseline models, metrics and statistical testing

This section focuses on two types of supervised machine learning models that can be trained using the provided labels: image classification and sequence classification tasks. The experiments serve two primary purposes: first, to establish a baseline for future research using the GastroHUN dataset, and second, to evaluate the complexity of categorizing the data. Performance for both tasks was assessed by weighted and macro precision, recall, and F1-score, metrics which account for class imbalances while assessing model performance. These metrics are defined as follows:

**Precision**, also known as Positive Predictive Value (PPV), is the ratio of correctly identified positive samples to all samples predicted as positive by the model. It measures the relevance of the retrieved positive instances:

1 $$precision=\frac{TP}{TP+FP}$$

**Recall**, also known as Sensitivity, True Positive Rate (TPR), is the ratio of correctly identified positive samples to all actual positive samples in the dataset. It measures how well the model captures all relevant positive instances:

2 $$precision=\frac{TP}{TP+FN}$$

**F1 score** is a measure of a model’s accuracy that combines both precision and recall into a single metric. It is calculated as the harmonic mean of precision and recall, providing a balanced assessment, especially in cases of imbalanced dataset:

3 $$precision=2\times \frac{precision\times recall}{precision+recall}$$

Model stability was evaluated by bootstrapping, using 100 iterations applied to the testing set^32^. At each iteration, 50% of the complete consensus-labeled samples for each patient were randomly and independently selected. Moreover, 95% confidence intervals were computed for each metric (e.g., precision, recall, F1-score) from the bootstrap results by calculating the sample mean ($\overline{x}$) and the margin of error. The margin of error was derived by first calculating the standard error of the mean (SEM), which is the sample standard deviation (*s*) divided by the square root of the bootstrap iterations (*b*). The SEM was then multiplied by the critical t-value (*t*_0.025_) corresponding to a 95% confidence level with *α* = 0.05. The margin of error was used to define the lower and upper bounds of the confidence interval, computed as follows:

4 $$\,{\rm{confidence\; interval}}\,=\overline{x}\pm \left({t}_{0.025}\times \frac{s}{\sqrt{b}}\right)$$

Where:

- $\overline{x}$ is the sample mean of the metric.
- *t*_0.025_ is the critical t-value for the two-tailed 95% confidence interval, given *α* = 0.05.
- *s* is the standard deviation.
- *b* is the number of bootstrap iterations (*b* = 100).

This methodology ensures a robust evaluation of model performance, accounting for variability in the testing set, and provides a clear measure of the model’s stability across different metrics. To ensure reproducibility, the code for these evaluations is available in the code repository.

#### Supervised image classification baseline

As mentioned, models were evaluated by partitioning data into 70% for training (270 cases), 15% for validation (58 cases), and 15% for testing (59 cases). Network architectures received 3 × *H* × *W* RGB images as input, being *H* and *W* the height and width, and each re-sized to 224 pixels using lanczos interpolation during pre-processing, basically by computing the mean and standard deviation from the training samples to normalize all data. To ensure a straightforward comparison, architectures were trained in two distinct phases: (a) Initially, there was a “warm-up” phase focused on training the classification layers, during which they were trained for 10 epochs with a constant learning rate. (b) After this warm-up phase, a fine-tuning phase targeted the final 40% of the feature layers. This fine-tuning was conducted over 100 epochs to optimize model’s performance, with early stopping if the validation F1-macro score did not improve for 10 consecutive epochs. Details of networks and training configuration are presented below:

- **Family architectures:** ConvNeXt^33^, ResNet^34^, VGG^35^ and VisionTransformer (ViT)^36^ in PyTorch implementation.
- **Pre-trainned weights:** ImageNet_V1.
- **Optimizer**: Adam.
- **Loss function:** Weighted cross-entropy for class imbalance.
- **Learning rate for warmup:** 0,001 with gamma = 0,1.
- **Learning rate for finetuning:** each parameter group by gamma every step size epoch.
- **Output Layer Neural Network:** 23 (22 for stomach classification + 1 for additional category).

The model achieving the highest F1-macro score during the validation phase was selected for evaluating the testing set. Each method was assessed using defined performance metrics. Given the variability of labels by raters, Table 4 outlines the different configurations used to validate models. For all cases, testing used the label with the highest kappa.

**Table 4:** Distribution of the imaging dataset based on inter-observer and per-annotator agreement levels.

Image Dataset Distribution					
Strategy	Training label	Team	Train	Valid	Test
Consensus	All	A & B	3,722	793	803
	Triple	A & B	5,228	1,103	803
	FG	A	4,244	918	803
	G	B	5,028	1,078	803
	FG1 - G1	A & B	4,940	1,064	803
	FG1 - G2	A & B	4,811	988	803
	FG2 - G1	A & B	4,553	982	803
	FG2 - G2	A & B	4,528	953	803
Annotator	FG(1,2) - G(1,2)	A & B	6,165	1,316	803
	Patients	—	270	58	59
	Percentage	—	70%	15%	15%

The table details the data splits, with the test set held constant across all approaches. “FG” refers to Fellow Gastroenterologists (Team A), and “G” to Gastroenterologists (Team B).

#### Supervised Sequence Classification Baseline

In this experiment, the sequence dataset was divided into training, validation, and testing sets, ensuring that cases with sequences were consistently assigned to the same subsets as in the image classification task. Table 5 provides the number of samples for each specific set. As in the image classification task, testing set consists of samples with complete agreement among the annotators. Classification of sequences applies two methods: a multi-layer gated recurrent unit (GRU) and a Transformer encoder block. Each token is obtained by embedding each frame with the ConvNeXt_Tiny classification model. Features from contiguous frames are concatenated into three-dimensional tensors, incorporating a sequence dimension for batch processing, a temporal window dimension to capture dependencies across frames, and a feature dimension for detailed characteristics within each frame. This structure enables processing and analysis of sequences.

**Table 5:** Distribution of annotator-level datasets for image and sequence classification.

Image Classification			Sequence Classification	
Set	Patients	Images	Patients	Sequences
Train	270	6,165	159	3,401
Valid	58	1,316	32	654
Test	59	803	32	394
Total	387	8,834	223	4,729

The table shows the number of patients and data splits for these tasks. Note the reduction in sequence cases due to the absence of recorded video procedures.

### Baseline results

The experimental setup includes two tasks: image classification and sequence classification. The outcomes were assessed across three scenarios:

**Scenario A (Image Classification):**

- Evaluate different state-of-the-art deep learning architectures on GastroHUN using labels with complete agreement (see Table 4).

**Scenario B (Image Classification):**

- Analyze model prediction confidence by using different levels of agreement to train and evaluate the models (see Table 4).

**Scenario C (Sequence Classification):**

- Evaluate the effectiveness of using sequential data to identify gastric regions.

### Image Classification with Complete Agreement Labels

**Fig. 5:**
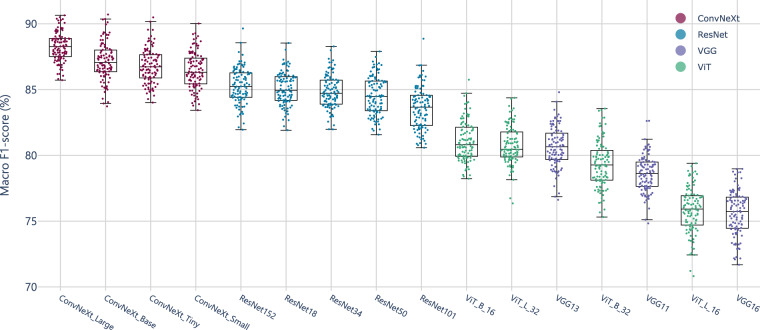
Bootstrap distribution of Macro F1-score rankings across different architectures, evaluated by repeated sampling (*b* = 100). Each point represents a ranking for a specific architecture obtained from a bootstrap iteration. The distribution of rankings shows the stability of each model’s performance with respect to sampling variability.

Sixteen models from four different architecture families were trained using samples with complete expert consensus about the labels (refer to the ‘All’ row in Table 4 for the ground truth). After training, models’ stability was evaluated by a bootstrap applied to the test set. As shown in Fig. 5, the distribution of macro F1-score rankings demonstrates that the ConvNeXt architecture family consistently outperformed other models across multiple evaluations.

Table 6 shows that ConvNeX_Large outperformed other models, demonstrating the highest stability and performance across all metrics, with a macro F1-score of 88.25 ± 0.22. Other ConvNeXt variants, such as ConvNeXt Base and ConvNeXt Tiny, also achieved competitive performance but showed slightly higher margins of error. Likewise, ResNet152 exhibited lower stability in top 5 model performance with a macro F1-score of 85.28 ± 0.27. Overall, ConvNeXt Large proved to be the most reliable model for handling multi-task challenges involving stomach anatomical landmarks.

**Table 6:** Top 5 performance metrics across bootstrap samples for different models.

model	macro			weighted		
	precision	recall	f1-score	precision	recall	f1-score
ConvNeXt_Large	88.83 ± 0.20	88.54 ± 0.23	88.25 ± 0.22	89.52 ± 0.19	88.71 ± 0.20	88.71 ± 0.20
ConvNeXt_Base	87.96 ± 0.27	87.53 ± 0.28	87.16 ± 0.29	88.72 ± 0.25	87.66 ± 0.27	87.64 ± 0.27
ConvNeXt_Tiny	87.58 ± 0.25	86.92 ± 0.27	86.79 ± 0.26	88.10 ± 0.24	87.28 ± 0.24	87.25 ± 0.25
ConvNeXt_Small	87.24 ± 0.27	86.77 ± 0.26	86.47 ± 0.28	87.82 ± 0.23	86.99 ± 0.26	86.90 ± 0.26
ResNet152	86.30 ± 0.26	85.49 ± 0.27	85.28 ± 0.27	86.82 ± 0.23	85.81 ± 0.25	85.76 ± 0.25

Macro and weighted metrics (precision, recall, and F1-score) are presented with their corresponding 95% confidence intervals (CIs), shown as “mean ± margin of error.” These results emphasize the robustness of each model’s performance, with ConvNeXt_Large exhibiting the highest stability and performance across all evaluated metrics.

**Fig. 6:**
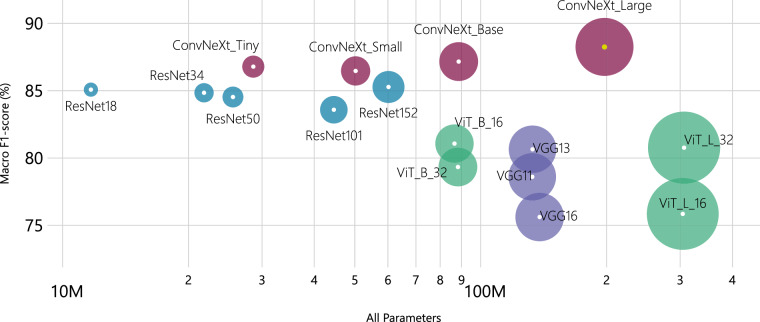
The bubble chart depicts the relationship between the mean bootstrap macro F1-score and the total number of parameters (including both feature extraction and classification layers) for each model.

Any model should balance performance and the number of parameters. Figure 6 visualizes this trade-off using a bubble chart, where each bubble’s size represents the number of parameters, and its position indicates the mean macro F1-score. The evaluation was comprehensive, with models trained on 3,722 images, validated on 793, and tested on 803, all with complete consensus. The results illustrate the relationship between model size and performance across different neural network architectures. ConvNeXt_Large achieves the highest F1-scores (88.25%) with 200M parameters, while lighter models like ResNet18 and ConvNeXt_Tiny reach  ~ 85% F1-score with only  ~ 11M and  ~ 28M parameters respectively. This suggests that some smaller architectures offer practical advantages where computational resources are limited.

#### Image Classification: Confusion matrix of ConvNeXt Large

**Fig. 7:**
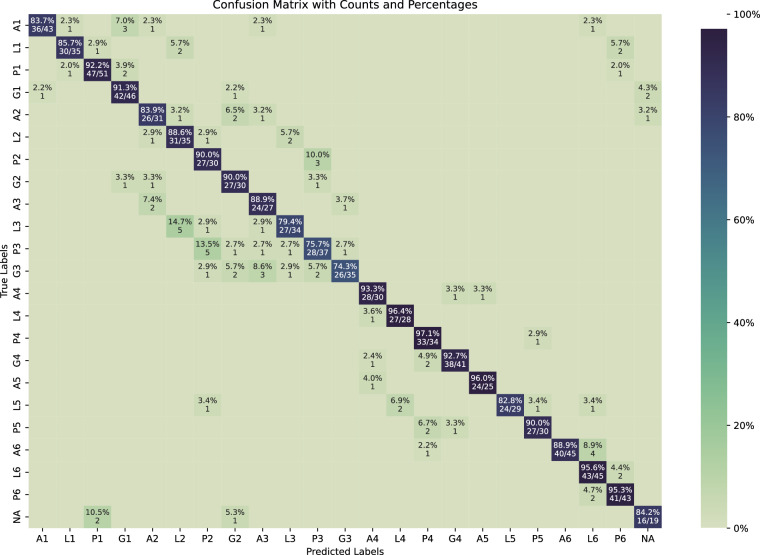
The confusion matrix for classifying images into 22 anatomical sites and an additional “not applicable” (NA) class.

The network achieved consistent performance in key regions like the antrum, lower body, and incisura (Fig. 7) but showed reduced accuracy in the middle body (L3, P3, and G3). While effective at detecting specific anatomical landmarks, challenges persist in classifying areas such as the cardia, lesser curvature, and posterior wall—regions where a higher rate of missed gastric cancer lesions has been reported in systematic reviews and meta-analyses^5,6,31^. Effective photodocumentation demands precise imaging, with standardized air insufflation and suction for better visibility. It is also worthy to note that testing results were exclusively obtained from images with complete agreement among four expert endoscopists. This consensus guarantees high-quality ground truth labels, but it misses a much more variable real-world scenario. Therefore, the future should focus on enhancing the model’s performance in situations when label variability or disagreements occur.

### Image Classification: Different Ground Truth consensus validation

**Fig. 8:**
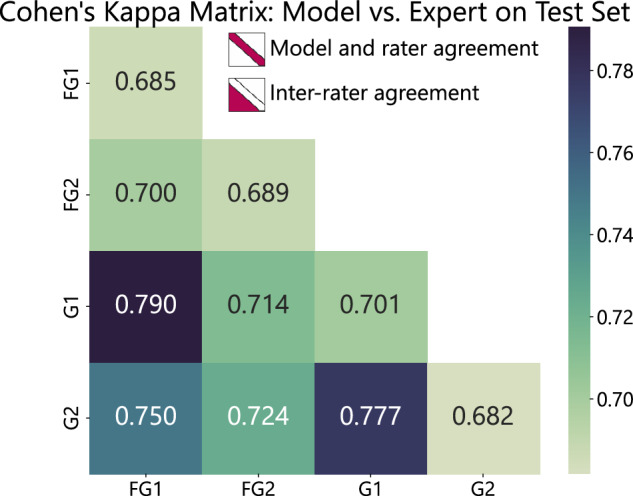
Cohen’s Kappa scores comparing model predictions with gastroenterologist labels across all samples in the test set, with the lower triangle showing agreement among experts ("FG” for Fellow Gastroenterologists, “G” for Gastroenterologists) and the diagonal representing model-expert annotations. The trained model was FG (consensus in Table 7).

In this experiment, models were trained independently based on varying agreement levels and annotators (see Table 4). The ConvNeXt_Tiny architecture was chosen for its parameter efficiency and macro F1 score in the model comparison (see Fig. 6). After training, the models’ stability was evaluated by a bootstrap applied to the test set. The results are shown in Table 7. In this validation, we present baseline metrics and analyze the dataset’s statistical characteristics to demonstrate its technical merit. The model trained with label agreements among Fellow Gastroenterologists (FG) achieved a macro F1-score of 87.05 ± 0.21, surpassing the best single annotator’s performance (G1) of 84.82 ± 0.23. Notably, this superior result was obtained with fewer training samples. Figure 8 displays Cohen’s kappa scores for the complete test set (including all samples without agreement) using the FG trained model. The diagonal shows the agreement between the model and each annotator, while the lower triangle illustrates the agreement among annotators. The highest model-annotator agreement was 0.701 (between the model and the G1 expert), while the strongest inter-annotator agreement was 0.790 (between G1 and FG1 experts). Given the extensive collection of images in GastroHUN, we invited fellow researchers to explore and develop innovative methodologies within the medical field. Recent advances in self-supervised learning and neural graph learning are promising for handling sparsely labeled or unlabeled data in image classification. Additionally, transformer-based architectures and contrastive learning techniques have shown effectiveness in improving feature extraction and classification accuracy. Multi-scale learning, which captures both fine and broad details, combined with advanced data augmentation techniques, can help build more robust models. These approaches can improve the ground truth labeling process, especially in cases with high inter-observer variability, and allow for a multilabel approach that leverages complex relationships within the data. We provide a baseline analysis and suggest future research using GastroHUN, focusing on advanced machine learning techniques to enhance image classification and address areas with high rates of missed lesions. Such advancements could enable researchers to comprehensively expand the dataset’s labeling, thereby enhancing its utility for future studies.

**Table 7:** Performance metrics by bootstrap: ConvNeXt_Tiny with consensus labels and individual annotators.

Strategy	Training label	macro			weighted		
		precision	recall	f1-score	precision	recall	f1-score
Consensus	All	87.58 ± 0.25	86.92 ± 0.27	86.79 ± 0.26	88.10 ± 0.24	87.28 ± 0.24	87.25 ± 0.25
	Triple	86.21 ± 0.27	85.15 ± 0.28	84.97 ± 0.28	86.66 ± 0.25	85.51 ± 0.27	85.41 ± 0.27
	**FG**	**88.11** ± **0.21**	**87.09** ± **0.21**	**87.05** ± **0.21**	**88.27** ± **0.19**	**87.43** ± **0.20**	**87.36** ± **0.20**
	G	86.42 ± 0.27	85.92 ± 0.27	85.66 ± 0.27	87.27 ± 0.23	86.40 ± 0.25	86.38 ± 0.25
	FG1 - G1	86.67 ± 0.27	85.49 ± 0.28	85.44 ± 0.28	86.85 ± 0.25	85.97 ± 0.27	85.87 ± 0.27
	FG1 - G2	86.92 ± 0.23	86.03 ± 0.24	85.94 ± 0.24	87.24 ± 0.22	86.37 ± 0.23	86.35 ± 0.23
	FG2 - G1	87.16 ± 0.28	86.14 ± 0.28	86.07 ± 0.28	87.89 ± 0.24	87.03 ± 0.26	86.93 ± 0.26
	FG2 - G2	85.61 ± 0.26	85.53 ± 0.27	85.11 ± 0.27	86.76 ± 0.26	85.99 ± 0.27	85.96 ± 0.27
Annotator	FG1	84.07 ± 0.27	82.88 ± 0.31	82.86 ± 0.30	84.75 ± 0.26	83.56 ± 0.27	83.63 ± 0.27
	FG2	85.37 ± 0.26	84.88 ± 0.27	84.33 ± 0.28	86.50 ± 0.24	85.27 ± 0.26	85.19 ± 0.26
	G1	85.64 ± 0.23	84.91 ± 0.23	84.82 ± 0.23	86.24 ± 0.22	85.39 ± 0.23	85.40 ± 0.23
	G2	84.21 ± 0.27	83.94 ± 0.26	83.53 ± 0.27	85.16 ± 0.23	84.07 ± 0.25	84.09 ± 0.25

Macro and weighted metrics (precision, recall, F1-score) shown with 95% confidence intervals. “FG” refers to Fellow Gastroenterologists (Team A), and “G” to Gastroenterologists (Team B).

### Sequence Classification: Performance metrics

The GRU and Transformer encoder were trained on the dataset using the frame embeddings generated by the ConvNeXt_Tiny model trained for image classification (Scenario B - see Table 7, FG-trained model). The analysis focused on a 23-frame temporal window (1.53 seconds) based on manual inspection to capture the largest possible sequence without including additional categories. The duration reflects the variability in the specialist’s observation time of a region, as observed during endoscopic photodocumentation. Performance in temporal scenarios was evaluated using macro precision, recall, and F1-score, with bootstrap applied to the complete agreement cases within the test set. Table 8 shows Transformer and GRU models performed similarly within the 23-frame window, with Transformer FG1-G2 achieving the highest F1-score (86.30 ± 0.42). Future work should explore optimal window sizes and self-supervised learning to improve multi-label sequence analysis, model generalization, and automatic photodocumentation of anatomical structures during endoscopic procedures.

**Table 8:** Comparison of macro precision, recall and f1-scores for sequence classification using transformer and GRU, utilizing a trained ConvNeXt Tiny model for sequence embedding.

Strategy	Training label	Transformer: macro			GRU: macro		
		precision	recall	f1-score	precision	recall	f1-score
Consensus	All	85.96 ± 0.47	86.34 ± 0.49	85.14 ± 0.48	85.49 ± 0.44	85.92 ± 0.44	84.86 ± 0.44
	Triple	81.46 ± 0.44	81.58 ± 0.45	80.51 ± 0.45	83.58 ± 0.44	83.17 ± 0.44	82.45 ± 0.43
	FG	85.31 ± 0.36	84.14 ± 0.39	83.33 ± 0.40	85.59 ± 0.40	84.40 ± 0.41	83.66 ± 0.41
	G	81.95 ± 0.45	81.34 ± 0.46	80.46 ± 0.45	86.74 ± 0.38	86.09 ± 0.39	85.47 ± 0.39
	FG1 - G1	86.21 ± 0.40	85.53 ± 0.45	84.81 ± 0.44	84.07 ± 0.44	83.27 ± 0.49	82.85 ± 0.47
	FG1 - G2	86.98 ± 0.42	87.01 ± 0.41	86.30 ± 0.42	86.15 ± 0.41	85.63 ± 0.39	85.01 ± 0.41
	FG2 - G1	83.83 ± 0.49	82.67 ± 0.49	82.03 ± 0.48	81.84 ± 0.50	81.52 ± 0.56	80.53 ± 0.51
	FG2 - G2	82.62 ± 0.42	83.77 ± 0.44	82.00 ± 0.44	78.38 ± 0.46	79.54 ± 0.45	77.53 ± 0.46
Annotator	FG1	80.99 ± 0.46	80.43 ± 0.49	79.52 ± 0.48	79.04 ± 0.53	78.40 ± 0.57	77.32 ± 0.56
	FG2	79.10 ± 0.45	79.35 ± 0.51	77.47 ± 0.44	76.79 ± 0.51	76.38 ± 0.58	74.37 ± 0.55
	G1	81.54 ± 0.44	80.68 ± 0.41	80.12 ± 0.39	82.03 ± 0.39	81.28 ± 0.44	80.59 ± 0.42
	G2	80.57 ± 0.52	80.27 ± 0.54	79.38 ± 0.51	78.67 ± 0.53	78.83 ± 0.57	77.53 ± 0.55

“FG” refers to Fellow Gastroenterologists (Team A), and “G” to Gastroenterologists (Team B).

## Usage Notes

The GratoHUN dataset is available at figshare^30^. To perform image or sequence classification experiments, we recommend using the scripts provided in our GitHub repository. Additionally, we have included all trained models in the README file on GitHub, along with Jupyter Notebook script for quick testing to obtain reproducible results.

## Data Availability

Alongside the data release, we are also providing access to the code utilized in our experiments. The complete code and any supplementary material needed for the experiments can be found on GitHub at https://github.com/Cimalab-unal/GastroHUN.git.
